# A Case of Eosinophilic Gastroenteritis Remission Achieved Steroid-Free With Mepolizumab

**DOI:** 10.1016/j.gastha.2022.03.003

**Published:** 2022-05-03

**Authors:** Yuki Hirata, Takahiko Nakamura, Kazuhide Higuchi

**Affiliations:** 1 Second Department of Internal Medicine, Osaka Medical and Pharmaceutical University, Takatsuki City, Japan; 2 First Department of Internal Medicine, Osaka Medical and Pharmaceutical University, Takatsuki City, Japan

**Keywords:** Eosinophilic gastroenteritis, Gastrointestinal endoscopy, Mepolizumab, Remission

## Abstract

Eosinophilic gastroenteritis is an eosinophilic inflammatory disease that responds well to steroids but often relapses upon their discontinuation, necessitating maintenance treatment. The anti–interleukin-5 antibody mepolizumab is useful for treating bronchial asthma and eosinophilic polyangiitis granulomatosa, and its efficacy in eosinophilic esophagitis has also been investigated. However, there are few reports of cases of eosinophilic gastroenteritis treated with mepolizumab. Here we report a case of steroid-dependent eosinophilic enteritis associated with asthma treated with mepolizumab that was maintained in steroid-free remission.

## Introduction

Eosinophilic gastrointestinal disorders are caused by the localized infiltration of eosinophils into the gastrointestinal tract, resulting in a variety of symptoms. They are broadly classified into eosinophilic esophagitis and eosinophilic gastroenteritis (EGE) according to the site of the affected gastrointestinal tract.^1^ EGE is often treated with systemic steroids, which are often very effective,^2^ but relapse commonly occurs upon their discontinuation.^3^ Therefore, small doses of steroids may be required as maintenance therapy despite the risk of side effects. Mepolizumab, a humanized monoclonal anti–interleukin (IL)-5 antibody, is effective in cases of severe asthma with a high eosinophil blood count.^4^^,^^5^ Here we describe a case of EGE associated with asthma that was maintained in steroid-free remission following treatment with mepolizumab, an anti–IL-5 antibody.

## Case Report

The patient was a 68-year-old woman who was treated as an outpatient for bronchial asthma and EGE. Upper gastrointestinal endoscopy at the time of diagnosis showed redness in the duodenum (Figure A), and biopsied tissue from the same area showed numerous eosinophilic infiltrates in the submucosa (Figure B). Computed tomography findings showed obvious wall thickening in the descending portion of the duodenum (Figure C). Capsule endoscopy revealed erythema and small erosions from the duodenum to the upper jejunum but almost no lesions in the ileum (Figure D). A blood allergy test showed allergic findings to cedar and Japanese cypress but no increase in specific immunoglobulin E to specific food antigens. The patient was started on 30 mg of systemic steroids, and the symptoms improved quickly; thus, the steroids were tapered off. Systemic steroid 5 mg/d was continued as maintenance therapy for EGE. When the dose of steroids was reduced to 2.5 mg/d, symptoms such as abdominal pain, nausea, and skin rash flared up immediately and the dose required another increase. Five doses of mepolizumab improved her fractional exhaled nitric oxide from 111 to 53 ppb and forced expiratory volume in 1 second from 75.8% to 81.4% and reduced the eosinophil fraction in the peripheral blood from 7.3% to 0.4%. Since no gastrointestinal symptoms were observed, the steroids were discontinued and an upper gastrointestinal endoscopy was performed. Eosinophilic infiltration in the submucosa of the duodenum was significantly decreased after mepolizumab treatment (Figure E and F). One year has passed since the start of mepolizumab treatment, and the patient is currently in clinical remission without systemic steroids and with no endoscopic or histological evidence of relapse (Figure G and H).

**Figure:**
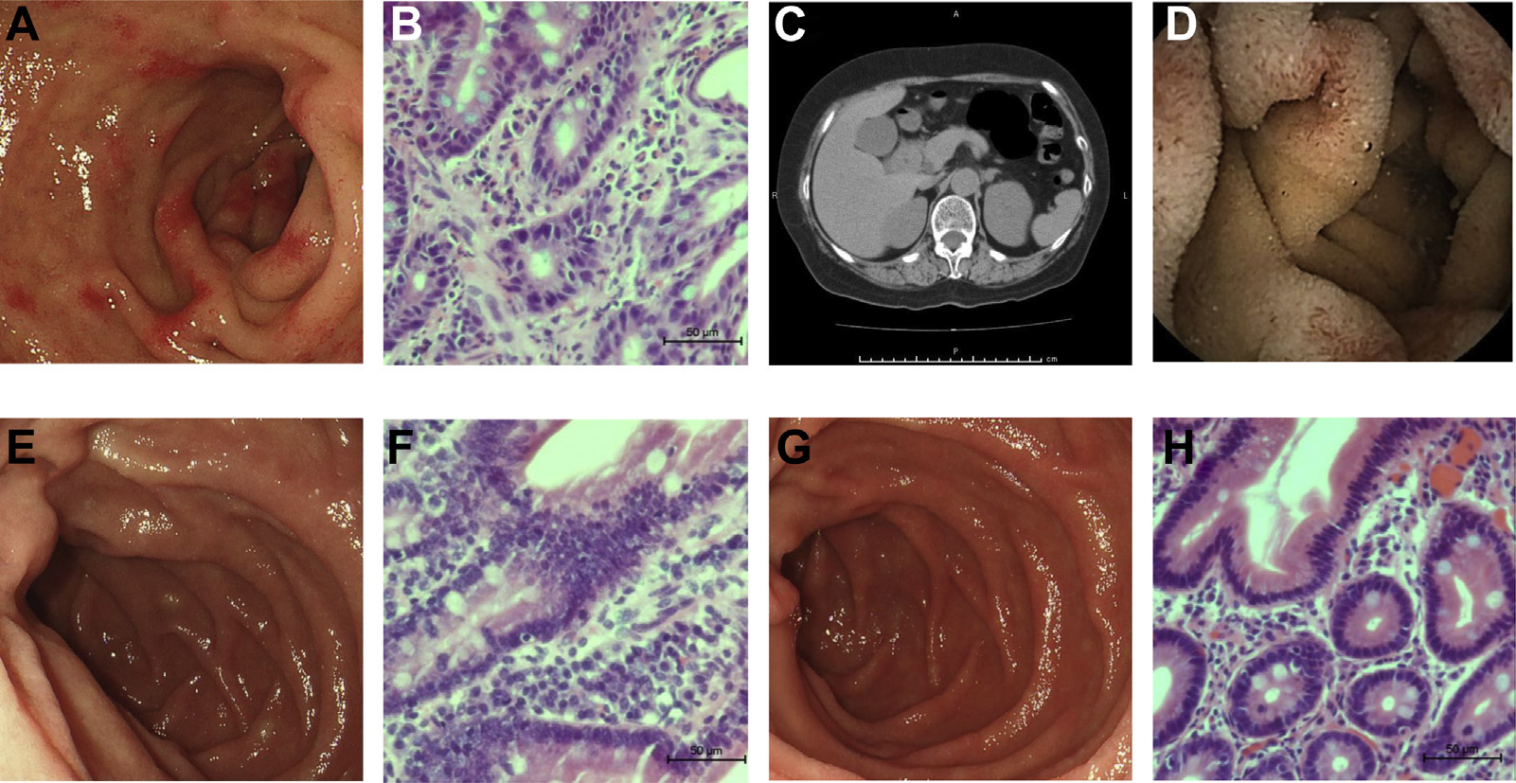
Upper endoscopic findings at the time of diagnosis showed scattered erythema in the duodenum (A). The pathological findings of the erythematous area showed numerous eosinophils infiltrating the submucosa (B). CT findings showed wall thickening in the descending portion of the duodenum (C). Capsule endoscopy at the time of diagnosis showed erythema and small erosions in the duodenum and upper jejunum (D). Both endoscopic and pathological findings after 5 doses of mepolizumab showed no obvious inflammatory findings (E and F). One year after the start of mepolizumab treatment, there was no obvious recurrence of inflammation on either endoscopic or pathological findings (G and H).

Informed consent was obtained from the patient for the publication of these images.

## Discussion

Most cases of EGE show symptomatic and histopathological improvement with systemic steroid administration. ^6^ On the other hand, there are cases in which the symptoms easily flare up when the dosage of steroids is reduced or discontinued.^7^ In such cases, remission cannot be maintained without maintenance use. However, the long-term administration of systemic steroids for EGE often leads to side effects such as osteoporosis, which is often problematic.^8^ IL-5 is involved in eosinophil differentiation and proliferation in bone marrow and prolonged survival and activation in the tissues, and its role in gastrointestinal diseases is becoming clearer. In ulcerative colitis, IL-5 expression is increased and histological images show eosinophil infiltration, suggesting that IL-5 is involved in the pathogenesis of the disease.^9^ In addition, IL-5–activated eosinophils are involved in IL-23–mediated chronic inflammation of the intestinal epithelium.^10^ The anti–IL-5 antibody mepolizumab has been shown to be clinically and histologically effective in the treatment of eosinophilic esophagitis.^11^^,^^12^ On the other hand, there are very few reports examining the effects of mepolizumab on EGE. In summary, this report showed that mepolizumab treatment of EGE associated with asthma enabled the maintenance of steroid-free remission. Thus, the administration of mepolizumab for EGE may be useful for safely maintaining remission.
